# Constitutive degradation of IκBα in human T lymphocytes is mediated by calpain

**DOI:** 10.1186/1742-4933-2-15

**Published:** 2005-11-04

**Authors:** Subramaniam Ponnappan, Sarah J Cullen, Usha Ponnappan

**Affiliations:** 1 Department of Geriatrics, University of Arkansas for Medical Sciences, Little Rock, AR, USA; 2 Department of Microbiology and Immunology, University of Arkansas for Medical Sciences, Little Rock, AR, USA; 3 VA Medical Research, Central Arkansas Veterans Health care system, Little Rock, AR, USA

## Abstract

**Background:**

Activation-induced induction of transcription factor NFκB in T lymphocytes is regulated by its inhibitor IκBα. NFκB activation has been demonstrated to occur either by phosphorylation on serine residues 32 and 36 of the inhibitor, IκBα, followed by ubiquitination and degradation of the inhibitor by the 26S proteasome, or by a proteasome-independent mechanism involving tyrosine phosphorylation, but not degradation. However, the mechanism underlying constitutive regulation of the levels of the inhibitor, IκB, in primary human T lymphocytes, remains to be fully delineated.

**Results:**

We demonstrate here, the involvement of a proteasome-independent pathway for constitutive regulation of IκBα levels in primary human T lymphocytes. Pretreatment with a cell permeable calpain inhibitor, E64D, but not with a proteasome specific inhibitor, lactacystin, blocks stimulus-independent IκBα degradation in primary human T cells. However, E64D pre-treatment fails to impact on IκBα levels following stimulation with either TNFα or pervanadate. Other isoforms of the inhibitor, IκBβ, and IκBγ, appear not to be subject to a similar ligand-independent regulation. Unlike the previously reported decline in ligand-induced degradation of IκBα in T cells from the elderly, constitutive degradation does not exhibit an age-associated decline, demonstrating proteasome-independent regulation of the activity.

**Conclusion:**

Our studies support a role for an E64D sensitive protease in regulating constitutive levels of IκBα in T cells, independent of the involvement of the 26S proteasome, and suggests a biological role for constitutive degradation of IκBα in T cells.

## Background

Transcription factor NFκB exists as homo-or-hetero-dimeric complexes, consisting of the Rel family of proteins [1]. These dimers operate as transcriptional regulators essential for a variety of cellular processes ranging from cell cycle progression to immune response gene induction [2]. In human T lymphocytes, like most other cells, NFκB exists in the cytoplasm coupled to its inhibitor IκBα or IκBβ, predominant members of IκB family of proteins [3]. A high affinity for RelA and c-Rel molecules enables these inhibitory proteins to associate with and thus restrict nuclear localization of the NFκB molecules. Most stimuli responsible for NFκB induction have been demonstrated to either invoke serine phosphorylation of the inhibitory proteins followed by ubiquitination and degradation via the 26S proteasome pathway, or involve the activation of tyrosine phosphorylation, as in the case of oxidative stress mediated stimuli, which is independent of proteasomal degradation mechanism [4,5]. While stimulation-induced modification of IκB has been studied extensively, little is known about the constitutive regulation of IκB protein in T cells under resting conditions.

Recent studies in B cell lines have demonstrated that IκBα, but not IκBβ, is constitutively degraded and is important for the induction of constitutive NFκB activity [6,7]. These studies indicate that constitutive degradation of IκB is mediated by a proteasome independent pathway. Studies also suggest that a calcium-dependent protease, calpain, may be important in regulating levels of IκBα [8-10]. These studies prompted us to investigate whether a similar regulation of "constitutive" i.e., stimulus-independent levels of IκBα occurs in primary T lymphocytes. Unlike B cells, NFκB induction and IκB regulation reported in T cells is clearly mediated by exogenous activating stimuli, with little or no constitutive nuclear NFκB present under basal condition.

Further, as NFκB regulation is significantly altered during aging in human T cells, we examined whether abnormal constitutive regulation may underlie lowered activation-mediated induction. Employing primary T cells obtained from human donors, we evaluated whether aging affects the regulation of constitutive levels of IκBα. We hypothesized that, as constitutive levels of IκBα were relatively unaffected by age in primary T cells, this may reflect minimal effect of age on calpain activity in T cells. We now report that E64D sensitive protease, calpain, is indeed responsible for regulating constitutive levels of IκBα, but not IκBβ or IκBγ, in human T cells. Further, calcium-ionophore mediated increase in calpain activity induced in T cells from young donors showed consistently higher activity at early time points after activation, when compared to the elderly. However, total calpain activity measured at the end of 60 minutes demonstrated no significant modulation based on the age of the donor. Thus, while the kinetics of calpain activation appears to be altered in T cells from the elderly, cumulative activity over a period of time remains unaffected. Additionally, we demonstrate that aging does not significantly affect stimulus-independent degradation of IκBα mediated by calpain, demonstrating proteasome-independent regulation. Thus, the calpain system is involved in the constitutive regulation of IκBα, and hence the NFκB signaling pathway, under resting conditions, in primary human T lymphocytes.

## Results

### Treatment with a cell permeable cysteine protease inhibitor, E64D, inhibits constitutive degradation of IκBα but not IκBβ or IκBγ in T lymphocytes from young and elderly donors

**Figure 1:**
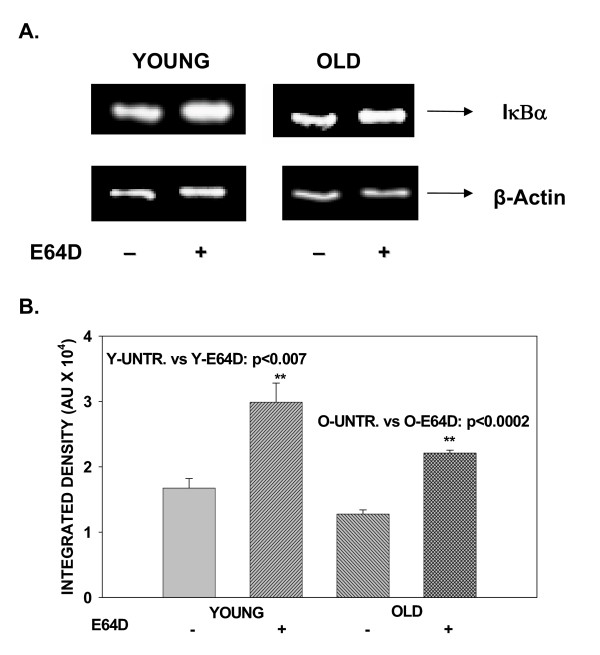
**Effect of treatment with E64D on constitutive levels of IκBα in T cells from young and elderly donors. **T cells obtained from young and elderly donors were either treated with E64D (+), 50 μM, or left untreated (-) for 45 minutes. At the end of incubation, cells were washed and cell lysates prepared. Lysates, equalized for protein, were resolved using SDS-PAGE, followed by electroblotting. Resolved proteins were detected using specific antibody to IκBα and ECL. Representative data from one donor pair are presented (A). Values obtained from a minimum of 4 donor pairs following densitometric scanning, are presented as mean integrated density units ± S.D.(B); ** indicates statistical difference from untreated controls.

**Figure 2:**
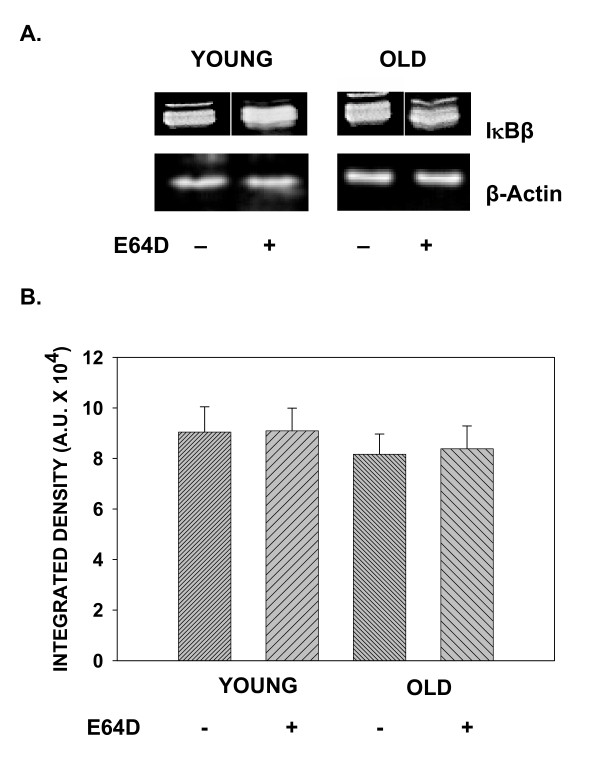
**Effect of age on constitutive degradation of IκBβ. **T cells obtained from young and elderly donors were either treated with E64D (+), 50 μM, or left untreated (-) for 45 minutes. At the end of incubation, cells were washed and cell lysates prepared. Lysates, equalized for protein, were resolved using SDS-PAGE, followed by electroblotting. Resolved proteins were detected using specific antibody to IκBβ and ECL. Representative data from one donor pair are presented (A). Values obtained from a minimum of 4 donor pairs following densitometric scanning, are presented as mean integrated density units ± S.D.(B).

**Figure 3:**
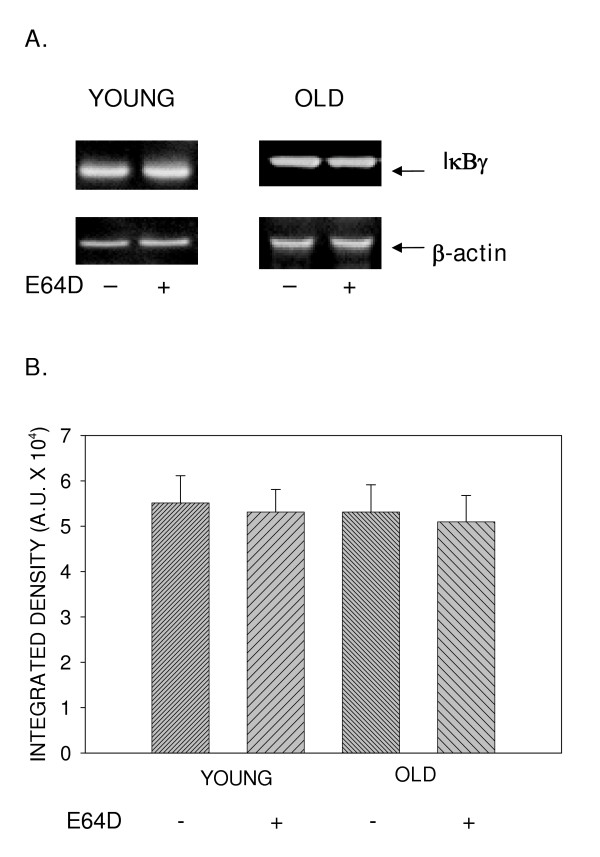
**Effect of age on constitutive degradation of IκBγ. **T cells obtained from young and elderly donors were either treated with E64D (+), 50 μM, or left untreated (-) for 45 minutes. At the end of incubation, cells were washed and cell lysates prepared. Lysates, equalized for protein, were resolved using SDS-PAGE, followed by electroblotting. Resolved proteins were detected using specific antibody to IκBγ and ECL. Representative data from one donor pair are presented (A). Values obtained from a minimum of 4 donor pairs following densitometric scanning, are presented as mean integrated density units ± S.D.(B).

We examined the effect of a cell permeable cysteine protease inhibitor, E64D, on the basal levels of IκBα, IκBβ and IκBγ, inhibitors of the ubiquitous transcription factor NFκB. Employing primary T cells and western blotting using antibody specific to the inhibitor isoforms, we now demonstrate that constitutive levels of IκBα, but not IκBβ or IκBγ are modulated by pretreatment with E64D. Results presented in fig. 1, demonstrate that pretreatment with E64D significantly inhibits constitutive degradation of IκBα with little or no effect on either IκBβ (fig. 2) or IκBγ (fig. 3). Levels of IκBα before treatment with E64D are significantly lower, than that observed following treatment, indicating inhibition of degradation.

As IκBα degradation induced by exogenous signals has been reported to be differentially regulated during aging [8,9], we next assessed the effect of treatment with E64D on IκB-α in T cells obtained from young and elderly donors. Results presented in fig. 1, demonstrate that, irrespective of the age of the T cell donor, E64D pretreatment significantly protected IκBα levels from constitutive degradation, and had little impact on isoforms, IκBβ (fig. 2) and IκBγ (fig. 3). Thus, aging does not influence cysteine protease-sensitive constitutive degradation of IκBα in T cells.

### Inhibition of cysteine protease activity by E64D does not affect TNFα-induced degradation of IκBα in T cells

**Figure 4:**
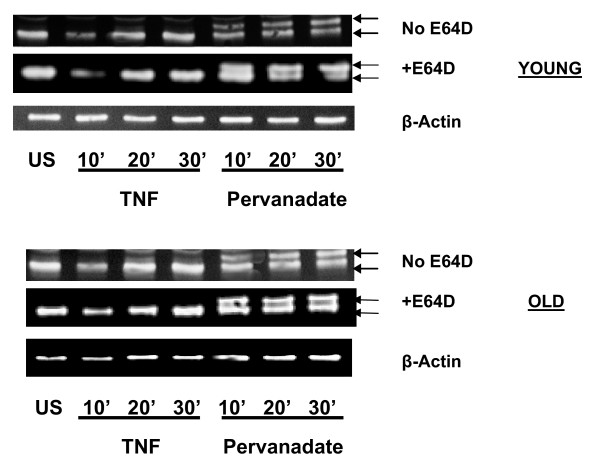
**Effect of E64D on stimulus-dependent modification of IκBα (Western Blots). **T cells obtained from young and elderly donors were either left untreated or treated with E64D (50 μM) for 45 min. At the end of incubation cells were stimulated with 100 μM pervanadate or TNFα (10 ng/ml) for 10, 20 or 30 min. Cell lysates were prepared and resolved by SDS-PAGE, using equal amounts of protein (30 μg / lane). Resolved proteins were transferred to nitrocellulose membrane and Western blotted with antibody to IκBα and detected using ECL. Representative results obtained from one donor pair are presented. Arrows represent IκBα specific bands detected and used in the analyses.

**Figure 5:**
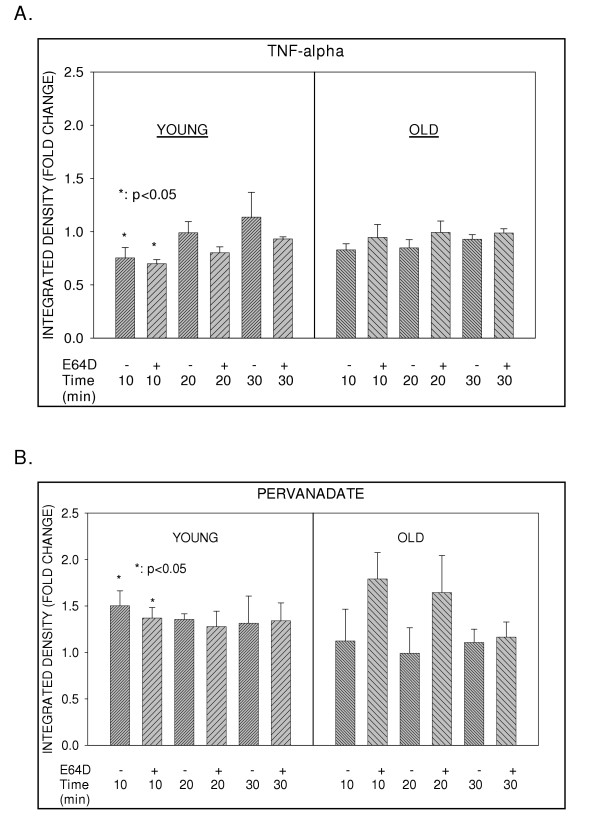
**Effect of E64D on stimulus-dependent modification of IκBα (Integrated Densities). **T cells obtained from young and elderly donors were either left untreated or treated with E64D (50 μM) for 45 min. At the end of incubation cells were stimulated with 100 μM pervanadate or TNFα (10 ng/ml) for 10, 20 or 30 min. Cell lysates were prepared and resolved by SDS-PAGE, using equal amounts of protein (30 μg / lane). Resolved proteins were transferred to nitrocellulose membrane and Western blotted with antibody to IκBα and detected using ECL. Mean integrated densities of IκBα specific bands obtained from a minimum of 4 donor pairs were used to determine fold change when compared to their respective controls and are presented as Mean (fold-change) ± S.D. [TNF-α (A); Pervanadate (B)].

Treatment of T cells by activating stimuli such as, anti-CD3 or TNFα, have been demonstrated to induce transcription factor NFκB activation by a signal-induced, proteasome-mediated degradation of the inhibitor IκBα. To determine whether such signal-induced degradation of IκBα was subjected to regulation by cysteine proteases, we next examined the role of E64D on activation-induced levels of IκBα. Results depicted in fig. 4 and 5A, clearly demonstrate that activation of T cells with TNFα induces degradation of IκB-α, irrespective of treatment with E64D. This indicates that E64D sensitive protease does not modulate or impact on activation-induced, proteasome-dependent degradation of IκBα. It should be noted that a slower mobility IκBα band appears at later time-points (20 and 30 min.) in TNF activated cells from the elderly. We believe that this represents modified IκBα. Future experiments will determine the precise nature of this modification. As proteasome dependent degradation of IκBα is clearly differentially regulated in T cells from young and elderly donors, we next examined, whether E64D mediated inhibition of constitutive levels impact differentially on the induced degradation of IκBα in T cells from the elderly. Results presented in fig. 5A, demonstrate that pre-treatment with E64D failed to influence activation-induced degradation of IκBα. Thus, irrespective of the age of the donor, treatment with E64D failed to modulate activation-induced degradation of IκBα.

### Pretreatment with E64D does not interfere with activation-induced modification mediated by pervanadate treatment in T cells

As a next step in the analyses, we examined the effect of pretreatment with E64D on modes of activation that does not involve signal-induced degradation of IκBα, such as those mediated by pervanadate. Similar to the observation with TNFα, pretreatment with E64D failed to impact on activation-induced IκBα modification in cells pre-treated with pervanadate, irrespective of the age of the donor (fig. 4 and 5B). It is important to note that IκBα in cells treated with pervanadate clearly demonstrate a slower mobility band representing tyrosine-phosphorylated IκBα. Thus, irrespective of the activation stimuli (TNFa or Pervanadate), E64D appeared not to impact on the activation-induced modulation of IκBα. NFκB dependent luciferase activity was also assayed following pervanadate treatment, in the presence or absence of E64D. In keeping with the data obtained with IκBα, E64D pretreatment, failed to impact on NFκB dependent luciferase activity, (data not shown).

### Treatment of T cells with proteasome inhibitor, Lactacystin, does not influence constitutive IκB-α levels, unlike that mediated by E64D

**Figure 6:**
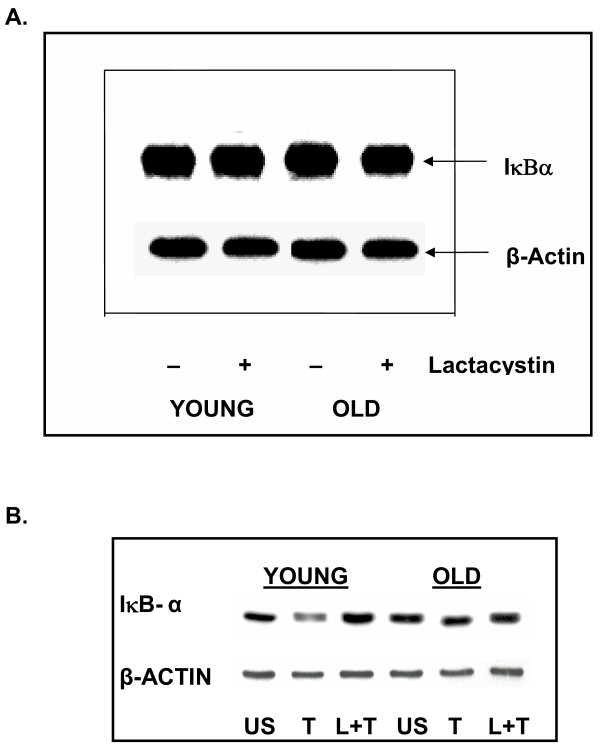
**Treatment with proteasome inhibitor, Lactacystin, fails to affect constitutive levels of IκBα in human T cells. **T cells obtained from young and elderly donors were either left untreated (-) or treated with lactacystin (5 μM) for 2 h to inhibit the proteasome. At the end of incubation cells were washed and lysates were prepared as described. Equal amounts of protein from cell lysates were resolved using SDS-PAGE and electroblotted. Resolved IκBα was detected using antibody to IκBα and ECL. To ensure equal protein loading, blots were stripped and reprobed with β-actin. Representative data from one donor pair are presented (A). As a positive control for lactacystin, T cells from young and elderly donors were either pretreated with Lactacystin (5 μM, 2 h) and then activated with TNFα for 10 min (L+T) or activated with TNFα without lactacystin pretreatment (T). US represent untreated T cells. Cell lysates were resolved using SDS-PAGE and IκBα detected as indicated above (B).

As IκB-α is constitutively degraded by E64D sensitive cysteine protease, we next assessed whether treatment with a proteasome inhibitor also interfered with this basal degradation. Results presented in fig. 6A, clearly demonstrate that pretreatment with lactacystin, a proteasome specific inhibitor, failed to influence basal levels of IκBα. Thus suggesting that basal or constitutive regulation of IκB-α is not dependent on the proteasome. To ensure that lactacystin did inhibit proteasome at the dose employed, i.e. positive control, T cells from young and elderly donors were either pretreated with lactacystin or left untreated. These cells were then subjected to treatment with TNFa. As seen in fig. 6B, TNFa treatment in the young induced degradation of IκBα, when compared to untreated controls. Pretreatment with lactacystin and then TNFα (L+T), inhibited TNFa mediated degradation. As reported previously, lactacystin is only minimally effective in T cells from the elderly.

### Treatment of T cell lysates with purified calpain, mimics constitutive degradation of IκBα

**Figure 7:**
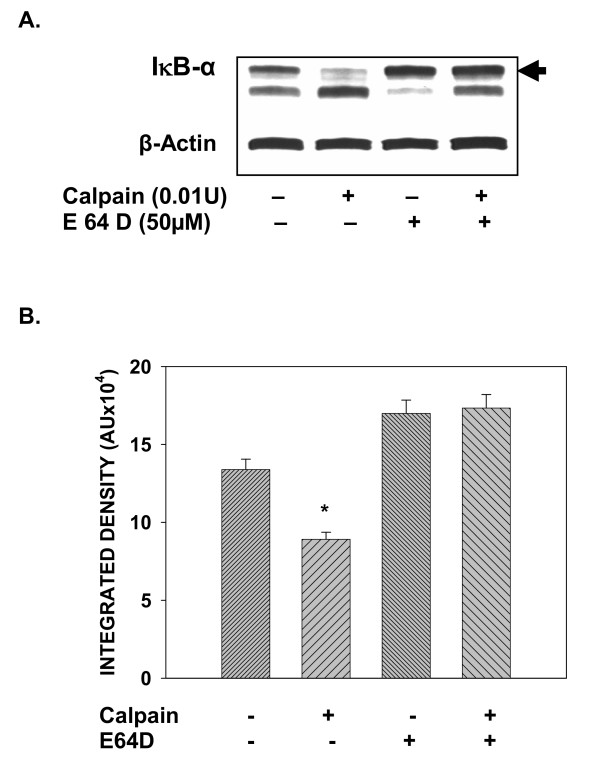
**Pretreatment with E64D inhibits calpain-mediated degradation of IκBα in T cell lysates. **T cell lysates obtained from young donors were pooled and 50 μg protein was incubated with 0.01 U purified calpain. The mixture was incubated at 37°C for 15 min. At the end of incubation samples were denatured and resolved using SDS-PAGE. IκBα was detected usingspecific antibody and ECL (A). Values obtained from a minimum of 4 donor pairs following densitometric scanning, are presented as mean integrated density units ± S.D. (B). * indicates significant degradation (p < 0.05).

As E64D pretreatment specifically inhibited basal degradation of IκBα, implicating a role for cysteine proteases such as calpain in regulating the constitutive levels of IκBα, we tested for a direct role for calpain by treating cytosolic lysates obtained from T cells with a purified preparation of calpain in an *in vitro *assay. T cell lysates equalized for protein from young donors were pooled and subjected to lysis in the presence of purified calpain, as indicated. Degradation of IκBα was determined by measuring the detectable levels of IκBα by SDS-PAGE and western blotting using antibody to IκBα. Results presented in fig. 7, show that upon exposure to 0.01 U of calpain, lysates from T cells demonstrate lowered levels of IκBα, indicating calpain-mediated degradation, which is inhibited by pretreatment with E64D.

### Kinetics of induction of calpain activity following calcium ionophore treatment, is modulated by age, but has no impact on overall effective calpain activity in T cells

**Figure 8:**
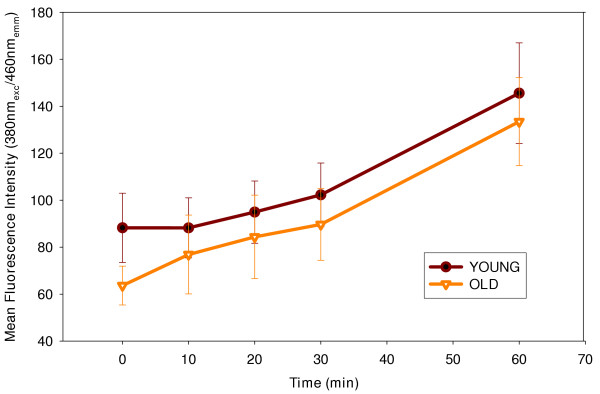
**Basal Calpain activity in T cells obtained from young and elderly donors. **T cells obtained from young and elderly donors were adjusted to 2.5 × 10^5 ^cells /ml. Calpain activity was measured using a fluorogenic substrate Boc-Met-AMC at 380 nm _exc _and 460 nm _emm _using a fluorometer. Specific activity was determined in the presence of calpain inhibitor E64D. Values represent calpain specific activity and represent Mean fluorescence intensity ± S.D of data obtained from 4 independent experiments.

To assess the effect of age on calpain activity, we obtained T cells from young and elderly healthy volunteers and examined them for endogenous calpain activity. As demonstrated in fig. 8, calpain specific protease hydrolyzing activity appeared to be slightly higher at time 0 in T cells from young donors; however, calcium ionophore induced increase in calpain activity was not significantly different between T cells from young and elderly donors. In fact, total hydrolyzing activity measured at the end of 60 minutes was not statistically different between cells obtained from the two age groups. Therefore, effective calpain activity remained unaffected by age of the T cell donor.

## Discussion

Calpain system has been demonstrated to be the main protease involved in constitutive degradation of IκBα [7-9]. Delineation of the exact region of IκBα necessary for degradation by calpain resulted in the identification of the C-terminal 39 amino acid sequence containing the PEST sequence to be critical for degradation in vitro by Shumway and Miyamoto [6]. However, their studies, demonstrated that calpain was not responsible for the degradation of IκBα in primary B cells [6]. Unlike B cells, that express constitutive nuclear NFκB, significant nuclear expression of NFκB in T cells occurs predominantly following stimulus-induced activation. Few studies, to date, have delved into constitutive regulation of NFκB in resting T cells. In our current studies, employing E64D, we demonstrate specificity of constitutive degradation of IκBα mediated by calpain in human primary T lymphocytes. The inhibition of this degradation in the presence of E64D, a cell permeable, cysteine protease inhibitor, supports the potential involvement of calpain activity in this process. Given the central role of NFκB in cell survival and signaling [11], constitutive degradation of the inhibitor IκBα is vital in understanding steady state kinetics of T cell regulation in the context of immune activation.

Our studies also demonstrate that degradation of IκBα under resting condition is refractory to proteasome inhibitor, Lactacystin, but not to calpain inhibitor, E64D. Therefore, unlike that reported for activation-induced degradation [12], constitutive levels of IκBα appear not to be subject to proteosomal regulation. This is particularly important given that our previous findings clearly showed that inducible degradation of IκBα is subject to an "age-effect" due to the inhibitory action of aging on proteasome- associated proteolytic activity [13,14].

Calpain-dependent degradation of IκBα has been demonstrated to occur in other cell types, which are refractory to proteasome activity [11]. Thus, it appears, that the degradation of IκBα can occur through two mutually exclusive pathways, dependent on the state of the cells, i.e., resting versus activated. Calpain system plays a role in constitutive, but not induced IκBα degradation, while proteasome degradation dictates induced levels in T cells [6,12]. Calpain activity has been demonstrated to be involved in the degradation of IκBα under certain conditions of viral infection [8]. It is therefore likely that this ability of constitutive degradation may be exploited by certain pathogens.

Unlike the most predominant inhibitor IκBα; IκBβ and IκBγ isoforms, appear not to be susceptible to this calpain-mediated degradation. Recent elegant experiments by Miyamoto et al implicate similar degradation kinetics for IκBα isoform in B cell lines [6]. Drawing upon the significance of such degradation events in the constitutive induction of NFκB in B cells, the role for constitutive regulation of NFκB by the calpain pathway in primary T cells was examined here. Results from these experiments clearly provide a biological basis for stimulus-independent degradation and its importance in the maintenance of NFκB in cell survival, which is not evident, unless challenged by stimuli capable of inducing apoptosis (data not shown).

Our studies on the effect of advancing age on constitutive degradation of IκBα, clearly implicate absence of any effect of donor age on the maintenance of E64D protease sensitive/calpain activity responsible for this degradation. Experiments conducted to determine the impact of aging on calpain activity clearly indicate that the effective activity is unaltered during aging. This is also reflected in the levels of IκBα in resting T cells from young and elderly donors, which are unaffected by age. Thus, despite loss in proteasome activity accompanying aging, calpain-mediated degradation of IκBα remains unaltered, demonstrating little or no role for the proteasomal regulation in calpain-mediated pathway that regulates IκBα levels. This observation is in keeping with earlier reports from our laboratory that demonstrated minimal effect of age on overall cellular proteolytic activity, especially, T cell chymotryptic activity [15]. It is also interesting to note that reports on calpain activity as a function of advancing age have been conflicting, with some demonstrating increased activity, [16,17] and others, decreased activity [18,19], however, these studies either used other cell types, employed exogenous substrates or cell lysates for the evaluation of the activity. Using a fluorogenic model substrate that is cell permeable, we now demonstrate that, ionomycin-inducible specific calpain activity, inhibitable by E64D, is unaffected; however, proteolytic activity observed in T cell lysates appeared to follow different kinetics in cells from the young than those observed in the elderly. Importantly, while 90% of the activity in cells from the young was clearly inhibitable by treatment with E64D, only 50% of the activity was inhibitable in cells from the elderly (data not shown).

While constitutive degradation of IκBα is clearly regulated by E64D sensitive calpain in T cells, activation-induced degradation, appears unaffected by pretreatment with E64D. Similarly, while activation-induced degradation of IκBα is sensitive to proteasome inhibition, constitutive degradation is unaffected by pretreatment with lactacystin, a proteasome inhibitor. Clearly, susceptibility of IκBα to degradation is not only dependent on the state of activation but also on the specificity of the protease. The physiologic significance of the degradation of the inhibitor clearly dictates induction of NFκB levels, and thus anti-apoptotic or survival ability, under uninduced conditions. Thus, regulation of IκBα levels in basal state of a cell is crucial and sets the stage for activation-induced survival signals. These results also indicate that the calpain pathway works independently of phosphorylation, since neither TNF nor pervanadate that induce serine and tyrosine phosphorylation, respectively, were affected by the inhibitor. Further, while proteasome dependent activation-induced degradation pathway, as well as proteasome pathway has been demonstrated to be compromised in T cells during aging, calpain activity clearly appears to be still functional, and is minimally affected by advancing age.

## Conclusion

In summary, we have demonstrated that basal levels of IκBα, but not IκBβ or IκBγ are subject to regulation by E64D sensitive protease, and can be mimicked by pretreatment with calpain. The regulation of IκBα levels by cysteine protease appears to have no effect on activation-induced IκBα or on other isoforms of IκB, irrespective of the stimuli employed. Additionally, it appears that interference with this decrease in basal degradation of IκBα does not impact on cell survival under resting conditions.

## Methods

### Materials

Fluorochrome labeled anti-CD3, and FITC- and PE-labeled isotype controls were obtained from Sigma Chemical Co. (St. Louis, MO). Anti IgG coupled to horseradish peroxidase was obtained from BD-Transduction Laboratories (Lexington, KY). All other antibodies were from Santa Cruz Biotech (Carlsbad,CA). Enhanced Chemi-luminescence reagents were from Amersham (Arlington Heights, IL). All fine chemicals unless otherwise mentioned were obtained from Sigma Chemical Company, (St. Louis, MO.), Electrophoresis supplies and Molecular weight standards were from BioRad (Richmond, CA.). E64D and lactacystin were from Calbiochem (CA). Substrate for Calpain was from Molecular probes, (Eugene, OR).

### Human subjects

Blood was obtained from healthy individuals living in the community. Young donors were between 21 and 30 years and old donors were between 65 and 85 years of age. A minimum of at least four donor pairs were used in each experiment. Both young and elderly donors were in good physical and mental health, had no apparent illness as suggested by an elaborate screening history and were not on any medication directly impacting the immune system during the course of this study.

### T Lymphocyte Isolation

Peripheral blood was obtained and T cells were purified and maintained in RPMI 1640 culture medium as previously described (20). Magnetic sorting by negative selection was used to isolate CD3^+ ^T cells. Purity of the isolated T cells was determined by flow cytometry using anti-CD3 conjugated to FITC. Populations were 90–95% pure. Treatment of T cells (20 × 10^6^cells/ml) with pervanadate (100 μM, freshly prepared before use) was carried out for indicated times at 37°C, before cell lysates were prepared. For experiments involving the use of inhibitor, cells were treated with E64D at 50 μM for 45 minutes.

### Western blotting

Cytosolic extracts for Western blotting were prepared by homogenization of cells in lysis buffer (1 mM Hepes, 10 mM KCl, 1.5 mM MgCl_2_, and 1 mM sodium orthovanadate, and 0.5% NP-40) (20). The following reagents were added to all buffers prior to their use: 0.5 mM dithiothreitol (DTT), 0.5 mM phenylmethylsulfonyl fluoride (PMSF), and 10 μg/ml each of aprotinin, leupeptin, and soybean trypsin inhibitor. Protein content of cytosolic extracts was determined using BioRad protein assay. Cell lysates equalized for protein (40 μg) were resolved by SDS-polyacrylamide gel electrophoresis (PAGE), transferred to nitrocellulose, immuno-blotted with specific antibody/s, and detected using anti-IgG coupled to horseradish peroxidase followed by Enhanced Chemi-luminescence (ECL). Where possible samples from young and elderly donors were resolved on the same gel, but in experiments where different treatments were analyzed, samples from young and the elderly were resolved on different gels, but were run simultaneously, to avoid inter and intra experimental variability. Resolution of samples on different gels did not influence the outcome of the results.

#### Calpain activity assays

Using a cell permeable fluorescent substrate Boc-Met-AMC, calpain activity within live cells was measured using a spectrofluorometer. Fluorescence was measured in cell suspension (2.5 × 10^5^cells/ml) following the addition of fluorophore, using the LS-50 model, Perkin-Elmer Spectrofluorometer. The fluorometer was equipped with a magnetic stirrer and warmed with recirculating water at 37°C using a pump. Fluorescence was measured using excitation and emission wavelengths of 380 and 460 nm, respectively. Values were obtained using a time drive mode, for up to 60 minutes.

#### Statistical analyses

Data were analyzed using student's t-test. Differences were considered significant, if p < 0.05.
